# Supplementary material for: The Change P82L in the Rift Valley Fever Virus NSs Protein Confers Attenuation in Mice
Source: Viruses. 2021 Mar 24;13(4):542. doi: 10.3390/v13040542 (PMC8064099; doi:10.3390/v13040542)
Supplement: Supplementary file 1 [file viruses-13-00542-s001.pdf]

A

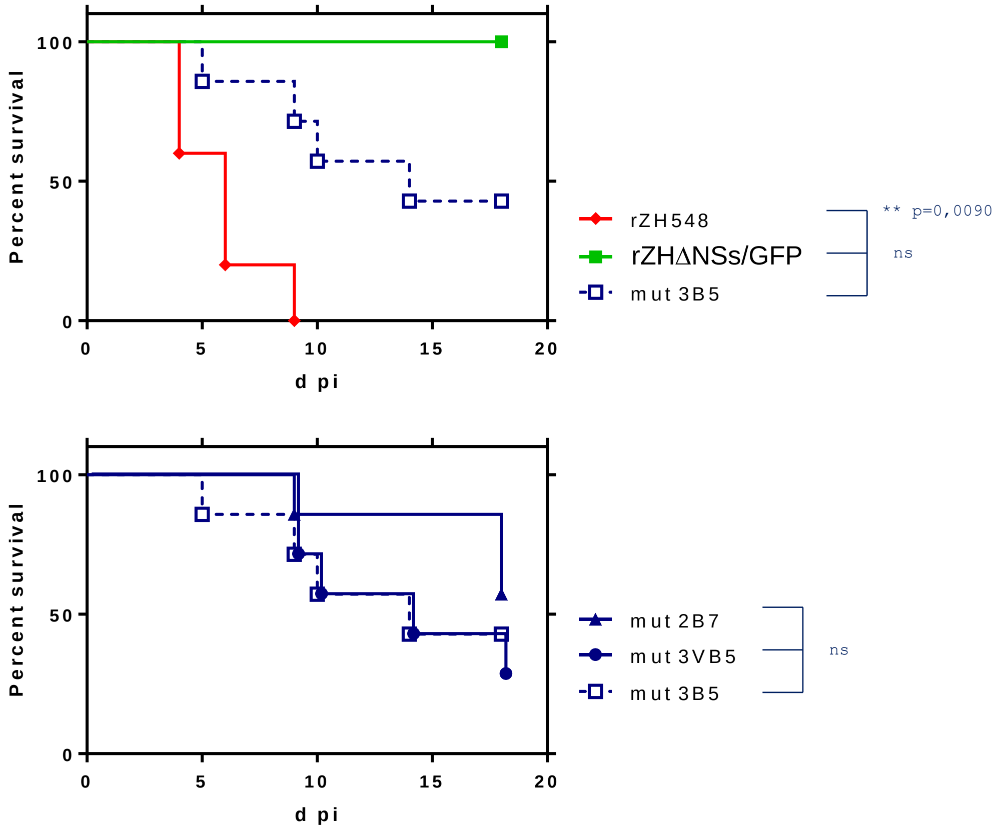

B

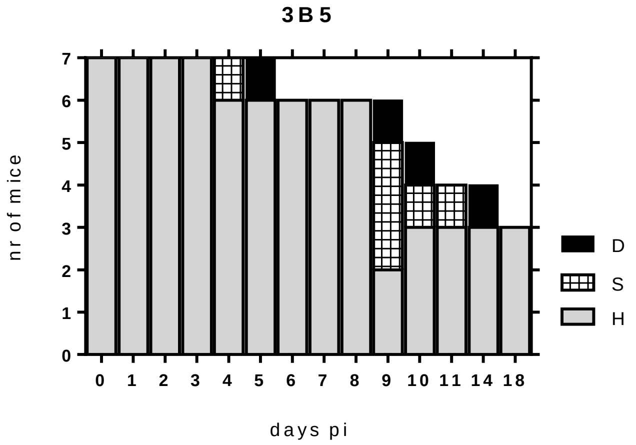

Supplemental Figure 1. (A) Survival plots of BALB/C mice upon challenge with rZH548-P82L mutant clone 3**B5.** Comparison with rZH548 and rZH548ΔNSs::GFP (upper plot) or mutant clones 2B7 and 3VB5 (bottom). Indicated p values according to the Log-rank (Mantel-Cox) test. (B) Morbidity upon challenge with mutant clone 3B5. The graph represents the clinical status of each mouse: D (dead/euthanized): black bars; S (signs-sick), hatched bars; H (healthy), grey bars.
